# Functional and Psychiatric Correlates of Comorbid Post-Traumatic Stress Disorder and Alcohol Use Disorder

**DOI:** 10.35946/arcr.v39.2.03

**Published:** 2018

**Authors:** Elizabeth Straus, Moira Haller, Robert C. Lyons, Sonya B. Norman

**Affiliations:** Elizabeth Straus, Ph.D., is a postdoctoral research fellow at VA San Diego Healthcare System and in the Department of Psychiatry, University of California San Diego School of Medicine, San Diego, California. Moira Haller, Ph.D., is a clinical psychologist at VA San Diego Healthcare System and an assistant clinical professor in the Department of Psychiatry, University of California San Diego School of Medicine, San Diego, California. Robert C. Lyons, M.S., is a graduate student in the Joint Doctoral Program in Clinical Psychology at San Diego State University and the University of California, San Diego, California. Sonya B. Norman, Ph.D., is a professor in the Department of Psychiatry, University of California San Diego School of Medicine, San Diego, California, and the director of the PTSD Consultation Program at the National Center for PTSD, White River Junction, Vermont

**Keywords:** alcohol use disorder, comorbidity, diagnostic criteria, post-traumatic stress disorder, psychosocial environment

## Abstract

Post-traumatic stress disorder (PTSD) and alcohol use disorder (AUD) are common comorbid conditions that affect large segments of the population. Individuals with comorbid PTSD/AUD face greater clinical and functional stressors than those with diagnoses of either PTSD or AUD alone. The purpose of this article is to review the phenomenology and functional associations of PTSD/AUD and address the common social, occupational, and psychological concerns associated with both disorders. Given the increased problems associated with comorbid PTSD/AUD, clinical and research efforts should focus on targeting functional and psychosocial problems in conjunction with psychiatric symptoms.

## Introduction

Post-traumatic stress disorder (PTSD) and alcohol use disorder (AUD) frequently co-occur. In the general population, approximately one-third of individuals with lifetime PTSD also meet criteria for lifetime AUD.^1^ In substance use treatment samples, up to two-thirds of those with AUD meet criteria for PTSD.^2^,^3^ Comorbid PTSD/AUD is associated with a more complex and severe profile than either disorder alone, including greater rates of having experienced childhood maltreatment, increased psychiatric comorbidities and reported symptom distress, decreased psychosocial functioning, and poorer prognosis.^1^,^4^

Despite the psychosocial impairment associated with PTSD/AUD, reviews on the comorbidity have largely focused on the clinical and neurobiological correlates associated with both disorders. Reviewing the psychosocial and functional burden of comorbid PTSD/AUD may improve understanding regarding the disorders and advance standards of care for a largely underserved population. The purpose of this review is to examine the clinical phenomenology, functional associations, and psychosocial factors associated with comorbid PTSD/AUD. Suggestions for future research and clinical practice are provided.

## Diagnostic Classifications of PTSD and AUD

According to the fifth edition of the *Diagnostic and Statistical Manual of Mental Disorders*, PTSD develops as a result of trauma exposure that included actual or threatened death, serious injury, or sexual violence (Criterion A).^5^ Common forms of trauma exposure include natural disasters, car accidents, combat, and physical or sexual abuse. Exposure must be either directly experienced, witnessed, learned about in the case of a close family or friend, or indirectly experienced in the course of one’s professional duties.

PTSD is characterized by four symptom clusters, which must be present for at least 1 month.^5^ The re-experiencing cluster (Criterion B) includes symptoms that are intrusive in nature and cause emotional or physiological reactivity (e.g., intrusive memories and psychological or physiological distress related to trauma reminders). Avoidance of internal or external trauma-related reminders (Criterion C; e.g., avoidance of memories, thoughts, people, or places associated with the traumatic event) is a prominent symptom cluster that contributes to the development and maintenance of PTSD. Negative alterations in cognition and mood (Criterion D) and alterations in arousal and reactivity (Criterion E) include exaggerated cognitive (e.g., negative beliefs about oneself, others, or the world), emotional (e.g., persistent negative emotional states and feelings of detachment or estrangement), and physiological responses (e.g., hypervigilance and problems with concentration) that appear or worsen after the traumatic event. In addition, the diagnosis requires that the symptoms cause either significant distress or functional impairment in social or occupational domains.

Symptoms of AUD fall within four domains:^5^

1. Impaired control (e.g., have had times when you ended up drinking more, or longer, than you intended)
2. Social impairment (e.g., continued to drink even though it was causing trouble with your family or friends)
3. Risky use (e.g., more than once have gotten into situations while or after drinking that increased your chances of getting hurt, such as driving, swimming, using machinery, walking in a dangerous area, or having unsafe sex)
4. Physical dependence (e.g., having to drink much more often than you once did to get the effect you want)

The diagnosis requires that at least 2 out of the 11 symptoms are met within the same 12-month period. The severity of impairment is based on the number of present symptoms (mild = 2 to 3, moderate = 4 to 5, or severe = 6 or more). Although diagnostically distinct, AUD and PTSD diagnoses share common psychosocial risk factors, and both result in impairments across multiple domains.

## Functional Associations Between PTSD and AUD

The high rates of comorbidity between PTSD and AUD necessitate the question of why these disorders frequently co-occur. Several causal mechanisms may link PTSD and AUD. (See the box **Functional Association Models** for a summary of these models.) First, the self-medication hypothesis posits that individuals use alcohol to cope with PTSD symptoms, such that PTSD causally influences risk for AUD. For instance, individuals with PTSD may use alcohol to improve sleep, irritability, or hypervigilance. Second, the high-risk hypothesis suggests that alcohol use may enhance the risk for PTSD by increasing the likelihood of trauma exposure or by impairing the detection of danger cues in the environment. Third, the susceptibility hypothesis theorizes that alcohol use may make individuals who have been exposed to trauma more vulnerable to its deleterious effects, thereby increasing risk for PTSD. AUD may increase susceptibility to PTSD by interfering with emotional processing following trauma exposure or by increasing anxiety or hyperarousal due to withdrawal symptoms.^6^ Finally, the shared vulnerability hypothesis posits that shared risk factors account for both PTSD and AUD, and their association is noncausal.

**Functional Association Models**

**Table:** 

Self-Medication	High Risk	Susceptibility	Shared Vulnerability
PTSD increases risk for AUD. Alcohol use is an attempt to reduce PTSD symptoms.	AUD increases risk for PTSD. Alcohol use impairs detection of danger cues in the environment. Alcohol use increases the risk of trauma exposure.	AUD increases risk for PTSD. Alcohol use interferes with emotional processing after exposure to trauma. Alcohol withdrawal symptoms increase anxiety and hyperarousal.	PTSD and AUD have similar risk factors and the association is noncausal. Risk factors can be: Genetic Environmental Individual (e.g., personality)

The self-medication hypothesis posits that having PTSD increases the risk for developing AUD, as individuals with PTSD may attempt to alleviate PTSD symptoms through the use of alcohol. A large body of evidence supports this hypothesis.^4^,^7^–^10^ For instance, data from a large, nationally representative sample demonstrated that using alcohol with the intent of reducing PTSD symptom distress was significantly associated with a lifetime history of AUD.^4^ Further, using longitudinal data from a community sample, Haller and Chassin found that PTSD symptoms predicted higher levels of later alcohol and drug problems, even when controlling for the effects of trauma exposure itself, pretrauma substance use, and pretrauma family risk factors that increase risk for both PTSD and AUD.^7^

Treatment studies also provide support for the self-medication hypothesis. For example, in a sample of women seeking treatment, improvement in PTSD symptom severity was associated with reduced substance use; however, substance use improvement was not related to decreased PTSD symptoms.^11^ These findings suggest that changes in PTSD symptoms may drive patterns of substance use, as posited by the self-medication hypothesis. Stewart and Conrod summarized the research on the association between both disorders by concluding that “PTSD has been shown to develop before the SUD [substance use disorder] in the large majority of comorbid cases in retrospective studies, and PTSD has been shown to increase risk for SUDs in prospective studies.”^12^^(p37)^

While several studies find support for a self-medication mechanism that may lead individuals with PTSD to develop drug and alcohol disorders,^13^,^14^ other studies specifically examining alcohol outcomes have failed to support a self-medication pathway causally linking PTSD to AUD.^15^ In a prospective longitudinal study of Persian Gulf War veterans, PTSD symptom clusters did not predict subsequent alcohol use concerns, although they did predict illicit drug use.^14^ Similarly, PTSD was not found to directly influence later problem drinking in a longitudinal study of women survivors of sexual assault.^15^ These studies reflect the complex relationship between PTSD and AUD and highlight the need to consider moderating factors and other mechanisms of risk. For instance, it may be that the functional association between PTSD and AUD varies based on both the form of trauma exposure and the type of substance use disorder.

Both the high-risk and susceptibility hypotheses suggest that AUD causally increases the risk for PTSD. Studies examining these hypotheses have generated mixed findings, with certain studies supporting only the high-risk hypothesis,^16^,^17^ others supporting only the susceptibility hypothesis,^18^ and some, when controlling for other risk factors, failing to support either hypothesis.^19^ Age and type of trauma may play a role in these mixed findings. At least two studies indicated that binge drinking^7^ and other high-risk behaviors (i.e., delinquent behavior, alcohol use, and drug use)^20^ during adolescence increased the likelihood of later exposure to assaultive violence (e.g., rape and physical assault), which carries an especially high risk for developing PTSD compared to other trauma types.^21^ Haller and Chassin found that although adolescent substance misuse conferred risk of exposure to assaultive violence, it did not increase the overall risk for trauma exposure.^7^ These findings suggest that alcohol use during adolescence may lead to PTSD as a result of the type of associated trauma exposure.

Shared environmental, genetic, and individual (e.g., personality) factors may also contribute to the overlap between PTSD and AUD in a noncausal manner. Behavioral genetic research indicates that heritable influences common to alcohol and drug use disorders account for 15.3% of PTSD variance,^22^ and genetic factors that contribute to trauma exposure and PTSD among women correlate (*r* = .54) with factors that contribute to AUD.^23^ Parental psychopathology and associated familial risk factors, such as family conflict/stress and exposure to childhood adversity, may also be shared risk factors for PTSD and AUD.^24^,^25^ Moreover, adverse childhood environments are associated with individual vulnerabilities and personality factors that may further increase risk for PTSD and AUD.

Relatedly, a variant of the shared vulnerability model—the trait vulnerability model—hypothesizes that PTSD symptoms may augment preexisting traits that confer risk for problems with alcohol. Multiple studies support this hypothesis.^26^,^27^ In particular, externalizing behavior (e.g., anger and aggression) appears to indirectly confer risk of both PTSD and AUD. In a community sample, PTSD was associated with an increase in early adulthood externalizing behavior that, in turn, was associated with alcohol misuse later in adulthood.^26^ Similarly, in a large sample of college students, PTSD was associated with increased disconstraint (i.e., the tendency to engage in risky or impulsive behavior), which was then associated with alcohol use problems.^27^

It is important to note that shared risk factors for PTSD and AUD may differ based on gender. For instance, in a study using a college sample, different facets of emotion regulation (e.g., problems controlling impulses and engaging in goal-directed behavior) for men and women were associated with PTSD and the alcohol-related consequences.^28^ In men, PTSD symptoms were related to increased impulse control difficulties, which, in turn, were associated with alcohol-related consequences. In women, PTSD was associated with difficulties engaging in goal-directed behavior, which, in turn, were associated with an increase in alcohol-related consequences. However, this study used a cross-sectional design, so it is not possible to infer a temporal association between the variables. Nonetheless, these findings underscore the need for models to account for the contribution of shared factors common to both PTSD and AUD, while also considering how such factors may vary based on gender.

Regardless of the causal mechanisms or shared factors responsible for the emergence of PTSD/AUD, once both disorders exist, it is possible that they mutually maintain and exacerbate one another (mutual maintenance model). For instance, alcohol may be used to attempt to suppress PTSD symptoms, but repeated use may interfere with natural recovery from trauma and also lead to physiological effects that heighten anxiety. As a result, PTSD symptoms and alcohol misuse may exert bidirectional influences on each other over time. A number of findings provide evidence of a bidirectional relationship between the disorders. For instance, in a sample of individuals seeking treatment for substance use disorder, avoidance symptoms (e.g., evading trauma-related reminders) were significantly elevated in patients with AUD, when compared to patients without AUD.^29^ The authors suggested that individuals with PTSD/AUD initially may have used alcohol in an attempt to alleviate avoidance symptoms, however, alcohol use could have subsequently exacerbated their avoidance. Further, in a sample of adults, PTSD symptoms predicted risk of AUD symptoms and vice versa, although the bidirectional relationship was stronger for women.^30^ Such findings are bolstered by the observations of individuals diagnosed with PTSD/AUD. Brown and colleagues found that patients with PTSD/AUD perceived the two disorders to be functionally related.^31^ These patients reported that when one disorder worsened, the other disorder was also more likely to worsen.

Although patient perceptions support the mutual maintenance model, empirical evidence regarding this model is mixed. In a recent longitudinal study, results indicated that PTSD symptoms led to alcohol misuse, but alcohol misuse did not appear to worsen the severity of PTSD over time.^32^ Prospective daily monitoring designs (measuring day-to-day symptom changes) provide a more nuanced method of examining comorbid disorders and the mutual maintenance model, but results from these studies are inconsistent. While some studies have shown partial support for both the mutual maintenance and self-medication models,^9^,^33^ another study supported only the self-medication hypothesis.^34^ Taking these mixed findings into account, Simpson and colleagues concluded that PTSD and AUD symptoms do influence one another (mutual maintenance model), but that PTSD appears to exert a greater influence on AUD symptoms (self-medication hypothesis), rather than the reverse.^9^

In summary, research suggests that there are multiple nonmutually exclusive pathways that underlie comorbid PTSD/AUD. Although the greatest body of evidence exists for the self-medication hypothesis, it is clear that common etiological risk factors also contribute to the comorbidity. Further, PTSD and AUD may have bidirectional influences on one another that serve to mutually maintain and exacerbate the symptoms of both disorders.

## Psychosocial Risk Factors

A substantial body of literature has demonstrated the association between having experienced childhood maltreatment (e.g., neglect or physical, sexual, or emotional abuse) and PTSD/AUD. Convergent findings suggest that biological and environmental determinants play a role in the comorbidity. For instance, neurobiological data suggest that childhood environmental stressors interact with genetic factors to contribute to the development of both disorders (see Brady and Back for a review).^35^ Moreover, individuals with co-occurring PTSD/AUD are more likely than those with PTSD or AUD alone to have experienced childhood maltreatment and other childhood environmental stressors.^1^

The heightened rate of childhood stressors in PTSD/AUD samples holds across diverse groups. In a nationally representative sample in the United States, individuals with comorbid PTSD/AUD had greater odds of having experienced childhood maltreatment (i.e., neglect or verbal, physical, or sexual abuse) and environmental stressors (i.e., vulnerable family environment, parental divorce, parental behavioral problems, or parental alcohol/drug problems) than individuals with either disorder alone.^1^ Similarly, in a small Austrian community sample, individuals with co-occurring PTSD/AUD were more likely to have experienced childhood sexual abuse (younger than age 16) or other adverse childhood stressors (e.g., growing up in the foster care system) than those who had PTSD only.^36^ Moreover, on average, those with PTSD/AUD were exposed to trauma a decade earlier than individuals with PTSD only. These findings suggest that childhood maltreatment and environmental stressors may lead to an increased risk of developing comorbid PTSD/AUD.

To add further support to this claim, a number of studies indicate that childhood maltreatment is associated with more severe and complex PTSD and AUD symptom profiles. Compared with trauma exposure during adolescence or adulthood, childhood maltreatment is associated with a longer course of PTSD,^37^ earlier onset of alcohol use and heaviest drinking periods,^38^ greater alcohol cravings in response to trauma cues,^39^ and increases in trauma-related symptom complexity (defined as the number of symptoms over a specified cutoff).^40^ The nature of childhood maltreatment also appears to uniquely affect psychiatric outcomes. In a sample of primary care patients in an urban community, greater childhood trauma exposure predicted higher PTSD total symptom severity scores, when controlling for level of adulthood trauma exposure.^41^ Furthermore, increases in childhood maltreatment exposure predicted greater alcohol use symptom severity, even when PTSD symptoms were held constant. Such findings may be explained, in part, by the characteristics of childhood maltreatment (e.g., chronic exposure perpetrated by attachment or authority figures) and the effects on the developing brain.^42^ Overall, the findings from these studies highlight the heightened rate and impact of childhood maltreatment for individuals who have PTSD/AUD.

## Psychosocial Outcomes

Comorbid PTSD/AUD is also associated with a range of deleterious mental health problems. A number of studies have demonstrated that in comparison to either disorder alone, co-occurring PTSD/AUD is associated with increased depression and anxiety, more severe PTSD and AUD symptoms,^1^,^43^,^44^ a greater likelihood of additional psychiatric comorbidities,^45^ and higher rates of suicide attempts.^1^,^4^ Given the severity of the mental health problems associated with co-occurring PTSD/AUD, it is not surprising that individuals with both diagnoses also experience psychosocial impairments across social, financial, and occupational domains.

Although the construct of social support is multidimensional and its association to trauma outcomes is varied,^46^ greater perceived social support likely serves as a protective factor against trauma-related disorders^47^ and is inversely associated with PTSD symptom severity^48^ and problematic alcohol use.^49^ The presence of PTSD and AUD, however, is associated with poorer social functioning. Although the existing literature has primarily focused on the relationship between social support variables and PTSD or AUD alone, a small body of work has investigated the social functioning deficits associated with comorbid PTSD/AUD.

In a study conducted by Riggs and colleagues, treatment-seeking individuals with comorbid PTSD/AUD were less likely to report living with a significant other (14%) than individuals with a single diagnosis of PTSD (42%) or AUD (56%).^50^ The specific pattern of social network problems was explored using a nationally representative sample in which individuals with comorbid PTSD/AUD were compared with those who had no psychopathology or who had either disorder alone.^51^ Individuals with comorbid PTSD/AUD experienced more problems with family support and apprehension (e.g., distress, discomfort, and anxiety) about engaging in close interpersonal relationships than individuals with either no diagnosis or a single diagnosis of PTSD or AUD.

The limited research on comorbid PTSD/AUD and functional impairments prompted Drapkin and colleagues to evaluate additional psychosocial factors (employment status, education level, income, and relationship status) across three samples of individuals seeking treatment.^52^ The samples consisted of individuals with comorbid PTSD/AUD, PTSD only, and AUD only. Interestingly, while comorbid PTSD/AUD was not associated with greater PTSD and AUD symptom severity (excluding alcohol craving), it was related to increased psychosocial impairment across multiple domains. Fewer individuals with co-occurring PTSD/AUD were employed or had a college education, when compared to those with either disorder alone. Furthermore, individuals with co-occurring PTSD/AUD were less likely than those with only PTSD or AUD to be living with a romantic partner. However, the authors noted that racial and gender differences across the groups could limit the validity of their results. While preliminary, these results suggest that both mental health and psychosocial deficits frequently affect individuals with comorbid PTSD/AUD.

## Clinical and Research Implications

Despite the many mental health and psychosocial problems associated with PTSD/AUD, a significant portion of individuals do not seek treatment for either disorder.^1^ Epidemiological studies reveal that only approximately one-quarter of individuals with AUD or PTSD diagnoses engage in disorder-specific treatment.^53^–^55^ Furthermore, when individuals with comorbid PTSD/AUD do initiate treatment, attrition rates are high and treatment effect sizes are small.

The literature discussed in this review highlights the many reasons why treatment retention and outcomes may be poor in this population. In particular, psychosocial concerns, including functional problems in social, educational, and occupational domains, disproportionately affect those with comorbid PTSD/AUD. Treatment studies with individuals who have comorbid PTSD/AUD have focused primarily on developing new treatments or modifying existing treatments to improve symptom outcomes. The findings of this review suggest that targeting functional problems and psychosocial stressors may help people with comorbid PTSD/AUD engage in treatments and achieve better outcomes. Multiple researchers^50^,^56^ have posited that the psychosocial factors associated with comorbid PTSD/AUD could partially account for the high attrition rates in randomized controlled trials, and that modifications to decrease psychosocial barriers to treatment may be critical. For instance, in a small study of veterans with comorbid PTSD and substance use disorder, all nine participants initiated and successfully completed prolonged exposure therapy while in a residential treatment program.^57^ Although the sample size was small, these preliminary findings highlight the potential of higher levels of care (e.g., intensive outpatient or residential treatment) to directly target psychosocial risk factors, such as decreased social support and housing issues, and, by doing so, improve PTSD treatment engagement. Further research is needed to examine the effectiveness of providing treatment for PTSD/AUD within higher levels of care.

Future research is also needed to continue to assess the relationship between key areas of psychosocial concerns and treatment outcomes in individuals with comorbid PTSD/AUD. Given the literature^55^ and current clinical practice guidelines put forth by the U.S. Department of Veterans Affairs and the American Psychological Association,^58^,^59^ which support the provision of trauma-focused treatments in comorbid populations, it will be important to continue to work toward improving initiation and completion of gold-standard treatments for PTSD among individuals with PTSD/AUD. The effectiveness of supplemental interventions designed to target nonclinical stressors (e.g., financial problems, occupational difficulties, and reduced social support) that might interfere with treatment engagement and completion should also be evaluated.

Such supplemental interventions may be designed and implemented at the program level (e.g., through higher levels of care, multidisciplinary models of care, or case management services) or at the individual level (e.g., through psychosocial assessments, gender-specific interventions, or developmental and patient-centered approaches to case conceptualization). Also, the delivery method for interventions may target the clinical and functional difficulties associated with comorbid PTSD/AUD. For instance, implementing interventions within a group context may bolster social support. Peer support programming, which emphasizes recovery-oriented and person-centered services, may facilitate positive social interaction and enhance individual self-efficacy within the treatment setting. Lastly, future research should examine whether preventive interventions designed to increase psychosocial resources are effective in reducing the likelihood of developing comorbid PTSD/AUD. For instance, enhancing engagement and functioning in social and occupational domains may protect against the development of PTSD/AUD.

## Conclusion

PTSD and AUD commonly co-occur and are associated with more complex and severe clinical presentations than either disorder alone. There are multiple etiological pathways that may influence the onset of comorbid PTSD/AUD and subsequently maintain and aggravate both disorders. Furthermore, comorbid PTSD/AUD is associated with more environmental risk factors, including a history of childhood maltreatment and functional problems (e.g., social and occupational concerns), than either disorder alone. Given the functional problems and low rates of treatment engagement and retention associated with PTSD/AUD, future research should evaluate the effect of psychosocial problems on treatment outcomes. Ultimately, an integrated model of care that focuses on both reducing symptoms and improving functional capacity across psychosocial domains may help improve treatment outcomes for this challenging clinical population.

## References

[1] Blanco C, Xu Y, Brady K (2013). Comorbidity of posttraumatic stress disorder with alcohol dependence among U.S. adults: Results from National Epidemiological Survey on Alcohol and Related Conditions. Drug Alcohol Depend.

[2] Gielen N, Havermans R, Tekelenburg M (2012). Prevalence of post-traumatic stress disorder among patients with substance use disorder: It is higher than clinicians think it is. Eur J Psychotraumatol.

[3] Seal KH, Cohen G, Waldrop A (2011). Substance use disorders in Iraq and Afghanistan veterans in VA healthcare, 2001–2010: Implications for screening, diagnosis and treatment. Drug Alcohol Depend.

[4] Leeies M, Pagura J, Sareen J (2010). The use of alcohol and drugs to self-medicate symptoms of posttraumatic stress disorder. Depress Anxiety.

[5] American Psychiatric Association (2013). Diagnostic and Statistical Manual of Mental Disorders.

[6] Kaysen D, Atkins DC, Moore SA (2011). Alcohol use, problems, and the course of posttraumatic stress disorder: A prospective study of female crime victims. J Dual Diagn.

[7] Haller M, Chassin L (2014). Risk pathways among traumatic stress, posttraumatic stress disorder symptoms, and alcohol and drug problems: A test of four hypotheses. Psychol Addict Behav.

[8] Ouimette P, Read JP, Wade M (2010). Modeling associations between posttraumatic stress symptoms and substance use. Addict Behav.

[9] Simpson TL, Stappenbeck CA, Luterek JA (2014). Drinking motives moderate daily relationships between PTSD symptoms and alcohol use. J Abnorm Psychol.

[10] Ullman SE, Relyea M, Peter-Hagene L (2013). Trauma histories, substance use coping, PTSD, and problem substance use among sexual assault victims. Addict Behav.

[11] Hien DA, Jiang H, Campbell AN (2010). Do treatment improvements in PTSD severity affect substance use outcomes? A secondary analysis from a randomized clinical trial in NIDA’s Clinical Trials Network. Am J Psychiatry.

[12] Stewart SH, Conrod PJ, Ouimette P, Brown PJ (2003). Psychosocial models of functional associations between posttraumatic stress disorder and substance use disorder. Trauma and Substance Abuse: Causes, Consequences, and Treatment of Comorbid Disorders.

[13] Breslau N, Davis GC, Schultz LR (2003). Posttraumatic stress disorder and the incidence of nicotine, alcohol, and other drug disorders in persons who have experienced trauma. Arch Gen Psychiatry.

[14] Shipherd JC, Stafford J, Tanner LR (2005). Predicting alcohol and drug abuse in Persian Gulf War veterans: What role do PTSD symptoms play?. Addict Behav.

[15] Najdowski CJ, Ullman SE (2009). Prospective effects of sexual victimization on PTSD and problem drinking. Addict Behav.

[16] Bromet E, Sonnega A, Kessler RC (1998). Risk factors for DSM-III-R posttraumatic stress disorder: Findings from the National Comorbidity Survey. Am J Epidemiol.

[17] Kilpatrick DG, Acierno R, Resnick HS (1997). A 2-year longitudinal analysis of the relationship between violent assault and substance use in women. J Consult Clin Psychol.

[18] Acierno R, Resnick H, Kilpatrick DG (1999). Risk factors for rape, physical assault, and posttraumatic stress disorder in women: Examination of differential multivariate relationships. J Anxiety Disord.

[19] Chilcoat HD, Breslau N (1998). Investigations of causal pathways between PTSD and drug use disorders. Addict Behav.

[20] Begle AM, Hanson RF, Danielson CK (2011). Longitudinal pathways of victimization, substance use, and delinquency: Findings from the National Survey of Adolescents. Addict Behav.

[21] Kessler RC, Sonnega A, Bromet E (1995). Posttraumatic stress disorder in the National Comorbidity Survey. Arch Gen Psychiatry.

[22] Xian H, Chantarujikapong SI, Scherrer JF (2000). Genetic and environmental influences on posttraumatic stress disorder, alcohol and drug dependence in twin pairs. Drug Alcohol Depend.

[23] Sartor CE, McCutcheon VV, Pommer NE (2011). Common genetic and environmental contributions to post-traumatic stress disorder and alcohol dependence in young women. Psychol Med.

[24] Koenen KC, Moffitt TE, Poulton R (2007). Early childhood factors associated with the development of post-traumatic stress disorder: Results from a longitudinal birth cohort. Psychol Med.

[25] Zhou Q, King KM, Chassin L (2006). The roles of familial alcoholism and adolescent family harmony in young adults’ substance dependence disorders: Mediated and moderated relations. J Abnorm Psychol.

[26] Haller M, Chassin L (2013). The influence of PTSD symptoms on alcohol and drug problems: Internalizing and externalizing pathways. Psychol Trauma.

[27] Read JP, Merrill JE, Griffin MJ (2014). Posttraumatic stress symptoms and alcohol problems: Self‐medication or trait vulnerability?. Am J Addict.

[28] Tripp JC, McDevitt-Murphy ME, Avery ML (2015). PTSD symptoms, emotion dysregulation, and alcohol-related consequences among college students with a trauma history. J Dual Diagn.

[29] Dworkin ER, Wanklyn S, Stasiewicz PR (2018). PTSD symptom presentation among people with alcohol and drug use disorders: Comparisons by substance of abuse. Addict Behav.

[30] Berenz EC, Roberson-Nay R, Latendresse SJ (2017). Posttraumatic stress disorder and alcohol dependence: Epidemiology and order of onset. Psychol Trauma.

[31] Brown PJ, Stout RL, Gannon-Rowley J (1998). Substance use disorder–PTSD comorbidity. Patients’ perceptions of symptom interplay and treatment issues. J Subst Abuse Treat.

[32] Langdon KJ, Fox AB, King LA (2016). Examination of the dynamic interplay between posttraumatic stress symptoms and alcohol misuse among combatexposed Operation Enduring Freedom (OEF)/Operation Iraqi Freedom (OIF) veterans. J Affect Disord.

[33] Possemato K, Maisto SA, Wade M (2015). Ecological momentary assessment of PTSD symptoms and alcohol use in combat veterans. Psychol Addict Behav.

[34] Hruska B, Pacella ML, George RL (2017). The association between daily PTSD symptom severity and alcohol-related outcomes in recent traumatic injury victims. Psychol Addict Behav.

[35] Brady KT, Back SE (2012). Childhood trauma, posttraumatic stress disorder, and alcohol dependence. Alcohol Res.

[36] Müller M, Vandeleur C, Rodgers S (2015). Childhood adversities as specific contributors to the co-occurrence of posttraumatic stress and alcohol use disorders. Psychiatry Res.

[37] Farrugia PL, Mills KL, Barrett E (2011). Childhood trauma among individuals with co-morbid substance use and posttraumatic stress disorder. Ment Health Subst Use.

[38] Waldrop AE, Ana EJ, Saladin ME (2007). Differences in early onset alcohol use and heavy drinking among persons with childhood and adulthood trauma. Am J Addict.

[39] Schumacher JA, Coffey SF, Stasiewicz PR (2006). Symptom severity, alcohol craving, and age of trauma onset in childhood and adolescent trauma survivors with comorbid alcohol dependence and posttraumatic stress disorder. Am J Addict.

[40] Cloitre M, Stolbach BC, Herman JL (2009). A developmental approach to complex PTSD: Childhood and adult cumulative trauma as predictors of symptom complexity. J Trauma Stress.

[41] Khoury L, Tang YL, Bradley B (2010). Substance use, childhood traumatic experience, and posttraumatic stress disorder in an urban civilian population. Depress Anxiety.

[42] U.S. Department of Health and Human Services, Administration for Children and Families, Administration on Children, Youth and Families, Children’s Bureau (2013). Child Maltreatment 2012.

[43] Rash CJ, Coffey SF, Baschnagel JS (2008). Psychometric properties of the IES-R in traumatized substance dependent individuals with and without PTSD. Addict Behav.

[44] Read JP, Brown PJ, Kahler CW (2004). Substance use and posttraumatic stress disorders: Symptom interplay and effects on outcome. Addict Behav.

[45] Ray LA, Capone C, Sheets E (2009). Posttraumatic stress disorder with and without alcohol use disorders: Diagnostic and clinical correlates in a psychiatric sample. Psychiatry Res.

[46] Robinaugh DJ, Marques L, Traeger LN (2011). Understanding the relationship of perceived social support to post-trauma cognitions and posttraumatic stress disorder. J Anxiety Disord.

[47] Brewin CR, Andrews B, Valentine JD (2000). Meta-analysis of risk factors for posttraumatic stress disorder in trauma-exposed adults. J Consult Clin Psychol.

[48] Ozer EJ, Best SR, Lipsey TL (2003). Predictors of posttraumatic stress disorder and symptoms in adults: A meta-analysis. Psychol Bull.

[49] Bravo AJ, Kelley ML, Hollis BF (2016). Social support, depressive symptoms, and hazardous alcohol use among Navy members: An examination of social support as a protective factor across deployment. J Soc Clin Psychol.

[50] Riggs DS, Rukstalis M, Volpicelli JR (2003). Demographic and social adjustment characteristics of patients with comorbid posttraumatic stress disorder and alcohol dependence: Potential pitfalls to PTSD treatment. Addict Behav.

[51] Dutton CE, Adams T, Bujarski S (2014). Posttraumatic stress disorder and alcohol dependence: Individual and combined associations with social network problems. J Anxiety Disord.

[52] Drapkin ML, Yusko D, Yasinski C (2011). Baseline functioning among individuals with posttraumatic stress disorder and alcohol dependence. J Subst Abuse Treat.

[53] Hasin DS, Stinson FS, Ogburn E (2007). Prevalence, correlates, disability, and comorbidity of DSM-IV alcohol abuse and dependence in the United States: Results from the National Epidemiologic Survey on Alcohol and Related Conditions. Arch Gen Psychiatry.

[54] Mackenzie CS, Reynolds K, Cairney J (2012). Disorder-specific mental health service use for mood and anxiety disorders: Associations with age, sex, and psychiatric comorbidity. Depress Anxiety.

[55] Roberts NP, Roberts PA, Jones N (2015). Psychological interventions for post-traumatic stress disorder and comorbid substance use disorder: A systematic review and meta-analysis. Clin Psychol Rev.

[56] Foa EB, Yusko DA, McLean CP (2013). Concurrent naltrexone and prolonged exposure therapy for patients with comorbid alcohol dependence and PTSD: A randomized clinical trial. JAMA.

[57] Norman SB, Davis BC, Colvonen PJ (2016). Prolonged exposure with veterans in a residential substance use treatment program. Cogn Behav Pract.

[58] U.S. Department of Veterans Affairs, U.S. Department of Defense (2017). VA/DOD Clinical Practice Guideline for the Management of Posttraumatic Stress Disorder and Acute Stress Reaction.

[59] American Psychological Association (2017). Clinical Practice Guideline for the Treatment of Posttraumatic Stress Disorder (PTSD) in Adults.

